# Severe Hypertriglyceridemia Without Pancreatitis Causing Total Pseudohypocalcemia With True Ionized Symptomatic Hypocalcemia: A Diagnostic Pitfall

**DOI:** 10.1002/ccr3.72629

**Published:** 2026-04-28

**Authors:** Mohamed Abuelazm, Marwah Algodi, Ahmed W. Hageen, Bassam Al Haly, Asawer Alsaeedi, Abdelrahman Awad, Hatem Eltaly, Omar Saab

**Affiliations:** ^1^ Faculty of Medicine Tanta University Tanta Egypt; ^2^ Jersey Shore University Medical Center Neptune City New Jersey USA; ^3^ Detroit Medical Center Detroit Michigan USA; ^4^ Baghdad University College of Medicine Baghdad Iraq; ^5^ Faculty of Medicine Mansoura University Mansoura Egypt; ^6^ Cleveland Clinic Main Campus Cleveland Ohio USA; ^7^ University of Arizona Tucson Arizona USA

**Keywords:** case report, hyperlipidemia, hypertriglyceridemia, pancreatitis, pseudohypocalcemia

## Abstract

Severe hypertriglyceridemia (HTG) can interfere with laboratory assays. HTG‐induced pancreatitis usually presents with hypocalcemia, but the effect of severe HTG, with no associated pancreatitis, on calcium levels is not well described. We present a 25‐year‐old male with severe HTG and diabetes who presented with generalized weakness, nausea, and vomiting. His labs revealed a severely elevated triglyceride level, as well as a critically low total calcium of 2.8 mg/dL, but only mildly reduced ionized calcium (1.10 mmol/L), with no evidence of laboratory or imaging findings of pancreatitis. Hypocalcemia signs were positive on physical examination. Treatment for HTG led to rapid normalization of total calcium levels, while intravenous calcium gluconate normalized ionized calcium and resulted in symptom resolution. This case shows that severe HTG can cause significant total pseudohypocalcemia alongside mild true ionized hypocalcemia, highlighting the importance of monitoring ionized calcium in this setting.

## Key Clinical Message

In severe hypertriglyceridemia, very low total calcium may reflect pseudohypocalcemia from assay interference, even without pancreatitis. Ionized calcium may be mildly reduced yet symptomatic. Relying on total calcium alone can cause misdiagnosis; ionized calcium should be measured in lipemic samples to guide appropriate management.

## 1 Introduction

Severe hypertriglyceridemia (HTG) is a well‐established cause of acute pancreatitis (AP), which can lead to true combined total and ionized hypocalcemia due to calcium deposition in the inflamed pancreatic tissue and disruption of calcium homeostasis. However, the effect of severe HTG on calcium levels in the absence of AP remains poorly described.

Severe HTG is a known cause of pre‐analytical interference in many biochemical tests. This interference primarily occurs through two mechanisms [1]. High lipoprotein levels can cause turbidity, which scatters light and leads to inaccurate total calcium measurements using spectrophotometric and colorimetric methods [2]. Also, the volume displacement effect can happen when a large amount of lipids shifts the plasma's aqueous phase. This results in an artificially low electrolyte measurement with indirect ion‐selective electrode methods, an effect observed for both sodium (pseudohyponatremia) and bicarbonate (pseudohypobicarbonatemia) [3, 4]. This can cause considerable diagnostic uncertainty, potentially leading to unnecessary tests and treatments due to artificially low electrolyte levels [4].

To the best of our knowledge, this case illustrates a rare presentation of severe total pseudohypocalcemia accompanied by mild true ionized hypocalcemia, caused solely by hypertriglyceridemia, in the absence of pancreatitis. It underscores a significant diagnostic challenge that can lead to misinterpretation of calcium status and inappropriate therapeutic interventions.

## 2 Case History/Examination

A 25‐year‐old male with a medical history significant for uncontrolled type 2 diabetes mellitus, hypertension, and HTG, complicated by medication non‐adherence, presented to the emergency department with a five‐day history of generalized weakness, accompanied by 2 days of nausea and non‐bilious emesis. He denied experiencing any abdominal pain, fever, or changes in bowel habits. However, he endorsed significant perioral numbness, as well as numbness in his hands and feet, accompanied by increased urinary frequency. The patient had a prior admission for diabetic ketoacidosis (DKA), but no history of pancreatitis episodes.

On examination, he was afebrile with normal vital signs, apart from mild tachycardia that subsequently resolved with IV fluids. The patient appeared lethargic with dry mucous membranes. A key finding was a positive Chvostek's sign on facial percussion. The remainder of his cardiopulmonary and abdominal examination was unremarkable.

## 3 Methods (Differential Diagnosis, Investigations and Treatment)

Key laboratory test results showed severe hyperglycemia without DKA; his glucose level was 574 mg/dL, with an HbA1c of 15%, and his serum beta‐hydroxybutyrate and anion gap were normal. The serum lipase was 56 U/L, and a computed tomography scan of the abdomen and pelvis revealed no evidence of acute pancreatitis.

**FIGURE 1:**
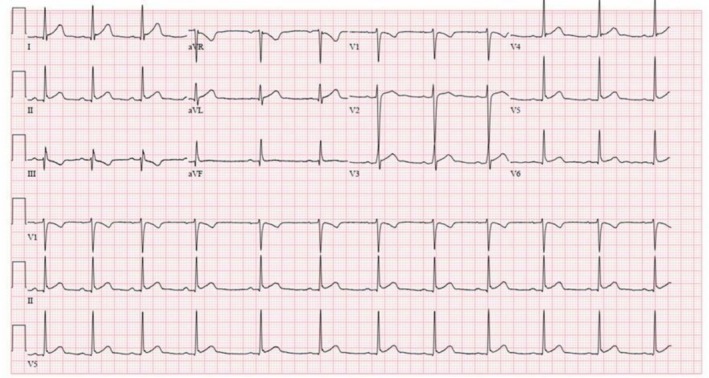
Electrocardiogram, showing a normal sinus rhythm with a corrected QT interval of 406 ms.

The most notable finding (Table 1) was a critically low total calcium level of 2.8 mg/dL (repeat 2.2 mg/dL), with a normal albumin level of 3.6 g/dL. In contrast, the arterial ionized calcium was only mildly reduced at 1.10 mmol/L (normal range: 1.15–1.30 mmol/L). His electrocardiogram (Figure 1) showed a normal sinus rhythm with a corrected QT interval of 406 ms. An initial measurable triglyceride level was 739 mg/dL; however, a subsequent measurement after plasma dilution exceeded 4425 mg/dL.

**TABLE 1:** Timeline of key laboratory values demonstrating pseudohypocalcemia and response to hypertriglyceridemia treatment.

Laboratory test	On admission	Follow‐up (Pre‐treatment)	(Post‐treatment)	Normal range
Calcium & lipids				
Total calcium (mg/dL)	2.2	2.8	7.8 then 8.2 then 9.1 then 9.4	8.6–10.3
Ionized calcium (mmol/L)	1.10	Not Measured	1.17	1.15–1.30
Triglycerides (mg/dL)	739*	> 4425	954	< 150
Metabolic Panel				
Glucose (mg/dL)	574	253	144	70–110
Sodium (mEq/L)	130	120	128	136–145
Organ function				
Lipase (U/L)	56	54	Not Measured	< 60
Albumin (g/dL)	3.6	2.9	3.9	3.5–5.5
Creatinine (mg/dL)	0.88	0.77	0.33	0.6–1.2
Endocrine & electrolytes				
Magnesium (mg/dL)	2.1	Not Measured	1.5	1.7–2.2
Phosphorus (mg/dL)	3.1	Not Measured	2.9	2.5–4.5
PTH, Intact (pg/mL)	37.8	Not Measured	Not Measured	15–65
Vit D, 25‐OH (ng/mL)	4	Not Measured	Not Measured	30–100
TSH (μIU/mL)	0.63	Not Measured	Not Measured	0.4–4
Venous pH	7.48	7.53	Not Measured	7.35–7.45
Bicarbonate (mmol/L)	24.3	25.1	27.1	22–29

Abbreviations: ED, Emergency Department; g/dL, grams per deciliter; mg/dL, milligrams per deciliter; MICU, Medical Intensive Care Unit; mmol/L, millimoles per liter; U/L, units per liter.

* This value likely represents a laboratory error that showed a falsely low level.

Secondary causes of hypocalcemia were evaluated. Magnesium (2.1 mg/dL) and phosphorus (2.7 mg/dL) were within normal limits. Renal tubular acidosis was ruled out by a venous blood gas showing alkalosis (pH 7.48) and normal bicarbonate levels (24.3 mmol/L). Notable findings included a severe Vitamin D deficiency (25‐OH Vitamin D: 4 ng/mL). However, the intact parathyroid hormone (PTH) level was unremarkably normal at 37.8 pg/mL. The absence of compensatory secondary hyperparathyroidism in the setting of Vitamin D deficiency and low total calcium strongly supports the diagnosis of pseudohypocalcemia rather than a physiological deficit.

## 4 Conclusion and Results (Outcome and Follow‐Up)

**FIGURE 2:**
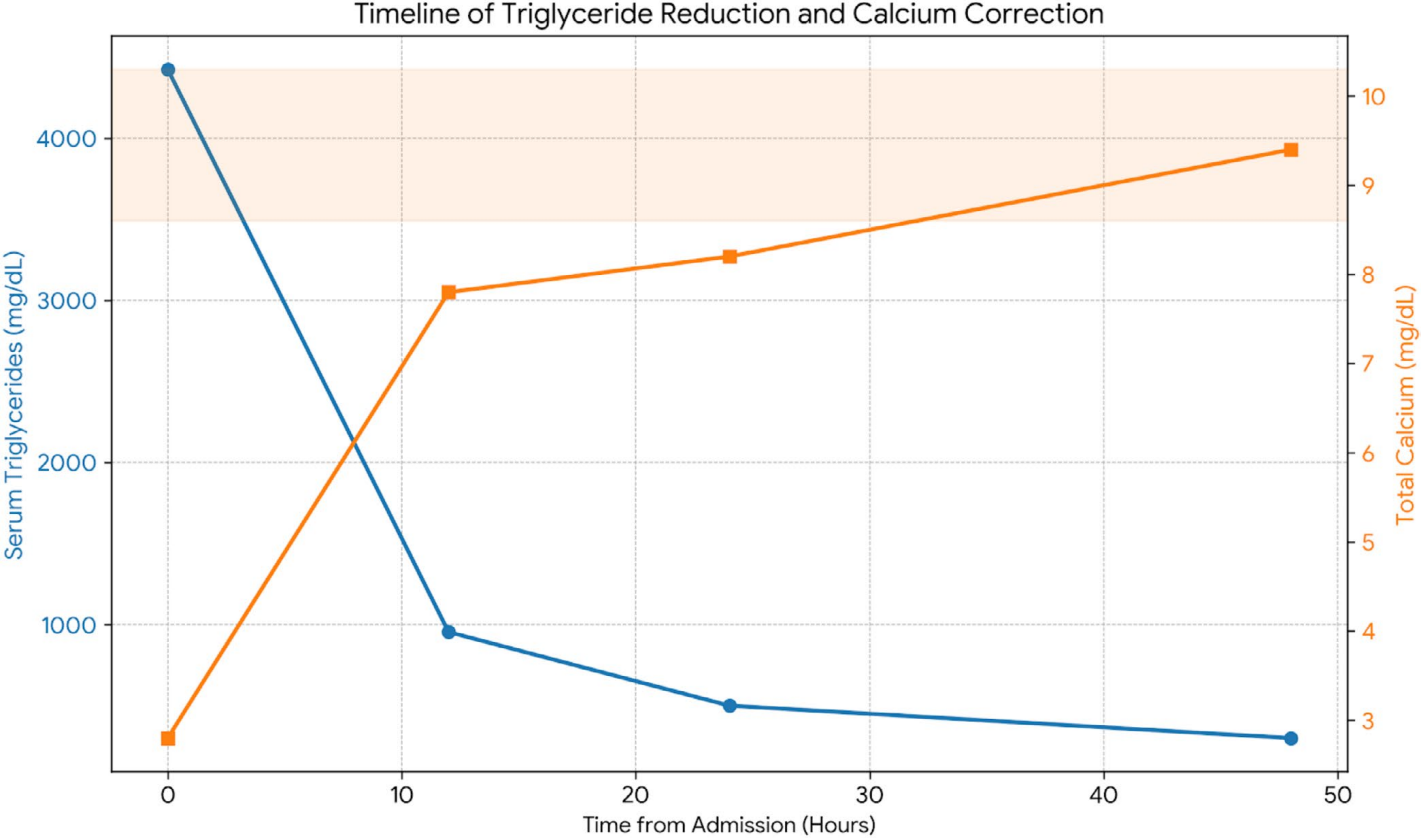
Timeline of Triglyceride and Calcium correction. The dual‐axis graph demonstrates the inverse relationship between serum triglycerides (blue line, left axis) and measured total calcium (orange line, right axis). At presentation (0 h), severe hypertriglyceridemia (> 4425 mg/dL) was associated with critically low measured total calcium (2.8 mg/dL). Following treatment with intravenous insulin and fluids, the rapid reduction in triglycerides coincided with the normalization of total calcium levels, confirming the diagnosis of pseudohypocalcemia due to assay interference. The shaded orange area represents the normal reference range for total calcium (8.6–10.3 mg/dL).

In the emergency department, the patient was treated with two liters of Lactated Ringer's and 2 g of intravenous calcium gluconate, as well as an intravenous insulin infusion for severe HTG. Following transfer to the intensive care unit, repeat labs a few hours later showed a dramatic increase in his total calcium to 7.8 mg/dL (Figure 2), with normalization of his ionized calcium to 1.17 mmol/L. Concurrently, his symptoms of perioral and extremity numbness resolved.

Our case demonstrates that severe HTG without pancreatitis can cause both total pseudohypocalcemia and mild true ionized hypocalcemia, leading to clinically significant symptoms. This observation challenges current evidence and highlights the importance of ionized calcium measurement in patients with dyslipidemia to avoid overcorrection of falsely low total calcium levels. Also, future studies are needed to understand the mechanisms linking TG metabolism to calcium regulation and to determine whether early detection could mitigate complications in similar presentations.

## 5 Discussion

Our patient presented with a critically low total calcium level of 2.8 mg/dL, accompanied by neuromuscular symptoms and a positive Chvostek's sign. The ionized calcium level was only mildly reduced (1.10 mmol/L), which suggests total‐pseudohypocalcemia with true ionized‐hypocalcemia. This could be explained by the interference in total calcium measurement in the setting of severe lipemia [2]. HTG is known to cause false reductions in electrolyte and analyte values in indirect ion‐selective electrode assays, owing to volume displacement and lipid‐induced turbidity. In such assays, plasma water content is underestimated, resulting in falsely low total calcium levels. This phenomenon was reported in a recent case with pseudohyponatremia caused by AP due to HTG [5].

Following treatment with intravenous calcium gluconate for symptomatic hypocalcemia and insulin infusion for HTG, total calcium increased markedly from 2.8 mg/dL to 7.8 mg/dL, then normalized. In contrast, ionized calcium returned to the normal range, from 1.10 mmol/L to 1.17 mmol/L. Clinically, the patient's symptoms resolved, supporting that the ionized fraction was physiologically relevant to his neuromuscular symptoms, as ionized hypocalcemia is most commonly associated with several manifestations, such as neuromuscular or cardiovascular insufficiency [6].

Still, a notable aspect of this case was the discrepancy between the severity of neuromuscular irritability (positive Chvostek's sign, significant paresthesias) and the relatively mild reduction in ionized calcium (1.10 mmol/L). This suggests that other metabolic factors lowered the symptomatic threshold for hypocalcemia—first, the patient presented with metabolic alkalosis (venous pH 7.48). Alkalosis promotes the binding of ionized calcium to albumin and directly increases neuronal excitability, thereby precipitating tetany even at calcium levels that might otherwise be asymptomatic [7]. Second, the rapid metabolic shifts associated with severe hyperglycemia (574 mg/dL) and osmotic diuresis may have created transmembrane electrolyte gradients that further destabilized nerve membrane potentials [8]. Accordingly, the aggregate effect of these rapid metabolic changes likely rendered the patient symptomatic despite a preserved absolute ionized calcium level.

The disproportionate increase in total calcium after a small dose of intravenous calcium gluconate (2 g, expected to raise total calcium by approximately 1 mg/dL) suggests that assay interference has been resolved due to the reduction in serum triglyceride concentration. This provides strong evidence that the initial total calcium measurement was significantly artifactually lowered, consistent with total pseudohypocalcemia, which can be explained by the volume displacement effect inherent to indirect ion‐selective electrode assays.

As described by Calmarza et al., the lipid phase in severe hypertriglyceridemia displaces the aqueous phase, leading to an artifactual underestimation of electrolytes such as calcium, which are dissolved only in the aqueous phase [2]. In contrast, the mechanism for the observed true ionized hypocalcemia remains hypothetical in the absence of pancreatitis. We can postulate that this may be driven by the intravascular sequestration of calcium by free fatty acids (FFAs). Older experimental models demonstrated that elevated FFAs can bind ionized calcium, forming insoluble calcium soaps, thereby inducing hypocalcemia in rats even in the absence of pancreatic inflammation [9, 10]. Similarly, in vitro studies have shown a dose‐dependent reduction in ionized calcium with increasing FFA concentrations [11]. It's worth noting that while these mechanisms appear plausible, they are derived from animal and in vitro studies. Therefore, further investigation is necessary to determine the extent to which this phenomenon occurs in humans solely with hypertriglyceridemia, compared with those also experiencing acute pancreatitis.

However, symptoms and low ionized calcium support the diagnosis of true ionized hypocalcemia. This raises the possibility of a true hypocalcemic state coexisting with laboratory investigations. This indicates that in certain individuals, even mild reductions in ionized calcium may precipitate significant neuromuscular symptoms [6, 12], particularly in those with underlying risk factors or metabolic dysregulation. Importantly, extracellular calcium concentrations are essential for the normal functioning of muscles and nerves.

The pathophysiological explanation for true ionized hypocalcemia in severe HTG without AP remains uncertain. One hypothesis is that elevated free fatty acid (FFAs) in severe HTG may bind and sequester ionized calcium, resulting in functional hypocalcemia even in the absence of AP‐induced lipolysis. This hypothesis is supported by both in vivo studies showing FFA‐induced reductions in serum calcium [9, 10] and in vitro human serum studies [11], demonstrating dose‐dependent decreases in ionized calcium with increased FFAs. Also, severe metabolic disturbances, such as poorly controlled diabetes, as evidenced in our patient by a blood glucose level of 574 mg/dL and HbA1c of 15%, may contribute to calcium imbalance through osmotic diuresis and renal electrolyte wasting [13].

This case highlights the importance of differentiating between artifactual and true hypocalcemia for management in patients with severe lipidaemia. Clinicians should avoid treating the numbers alone, as aggressive calcium supplementation based on a falsely low total calcium can precipitate iatrogenic hypercalcemia and soft‐tissue calcification. Calcium replacement should be reserved strictly for patients with documented low ionized calcium or frank neuromuscular symptoms. Therefore, we suggest that ionized calcium measurement should be conducted for any patient presenting with visible lipemia or serum triglycerides exceeding 1000 mg/dL, regardless of the reported total calcium level. Reliance on total calcium in this setting risks misdiagnosis and potential harm. Accordingly, in asymptomatic patients with isolated total hypocalcemia and severe hypertriglyceridemia, the primary intervention should be rapid triglyceride reduction, which resolves assay interference and normalizes total calcium without the need for supplementation.

## Author Contributions

**Mohamed Abuelazm:** conceptualization, data curation, investigation, supervision, writing – original draft, writing – review and editing. **Marwah Algodi:** data curation, investigation, methodology, writing – original draft. **Ahmed W. Hageen:** data curation, investigation, validation, writing – original draft. **Bassam Al Haly:** data curation, investigation, visualization, writing – original draft. **Abdelrahman Awad:** data curation, validation, writing – original draft. **Asawer Alsaeedi:** investigation, validation, writing – original draft. **Hatem Eltaly:** conceptualization, data curation, validation, writing – original draft, writing – review and editing. **Omar Saab:** conceptualization, supervision, validation, writing – original draft, writing – review and editing.

## Funding

The authors have nothing to report.

## Consent

Written informed consent was obtained for this case report.

## Conflicts of Interest

The authors declare no conflicts of interest.

## Data Availability

Data can be provided by the corresponding author upon reasonable request.

## References

[1] N. N. N. Andrade , M. V. Oliveira , and C. L. Souza , “Procedures to Minimize Interference of Hypertriglyceridemia in Laboratory Exams of Lipemic Samples in Acute Pancreatitis: A Case Report,” Jornal Brasileiro de Patologia e Medicina Laboratorial 52, no. 2 (2016): 103–106.

[2] P. Calmarza and J. Cordero , “Lipemia Interferences in Routine Clinical Biochemical Tests,” Biochemia Medica 21, no. 2 (2011): 160–166. 22135856 10.11613/bm.2011.025

[3] F. Aziz , R. Sam , S. Q. Lew , et al., “Pseudohyponatremia: Mechanism, Diagnosis, Clinical Associations and Management,” Journal of Clinical Medicine 12, no. 12 (2023): 4076. 37373769 10.3390/jcm12124076 PMC10299669

[4] S. Roy , M. Ashraf , S. S. Kang , R. Ayala , and S. Adapa , “Severe Hypertriglyceridaemia Leading to Factitious Hypobicarbonataemia,” European Journal of Case Reports in Internal Medicine 8, no. 12 (2021), 10.12890/2021_003046. PMC8765681 35059342

[5] R. S. Hansen , J. Revsholm , M. Motawea , and L. Folkestad , “Pseudohyponatraemia Caused by Acute Pancreatitis‐Derived Hypertriglyceridaemia,” BML Case Reports 14, no. 4 (2021): e241806. 10.1136/bcr-2021-241806 PMC8057565 33875511

[6] G. P. Zaloga , “Hypocalcemia in Critically Ill Patients,” Critical Care Medicine 20, no. 2 (1992): 251–262. 1737459 10.1097/00003246-199202000-00014

[7] J. Thode , N. Fogh‐Andersen , P. D. Wimberley , A. M. Sørensen , and O. Siggaard‐Andersen , “Relation Between pH and Ionized Calcium In Vitro and In Vivo in Man,” Scandinavian Journal of Clinical and Laboratory Investigation 43 (1983): 79–82. 6578581

[8] K. Dhatariya , O. Mustafa , and D. Stathi , “Hyperglycemic Crises,” Acute Endocrinology (2025): 119–147.

[9] M. A. Dettelbach , L. J. Deftos , and A. F. Stewart , “Intraperitoneal Free Fatty Acids Induce Severe Hypocalcemia in Rats: A Model for the Hypocalcemia of Pancreatitis,” Journal of Bone and Mineral Research 5, no. 12 (1990): 1249–1255. 2075838 10.1002/jbmr.5650051210

[10] A. L. Warshaw , K. H. Lee , T. W. Napier , P. O. Fournier , D. Duchainey , and L. Axelrod , “Depression of Serum Calcium by Increased Plasma Free Fatty Acids in the Rat: A Mechanism for Hypocalcemia in Acute Pancreatitis,” Gastroenterology 89, no. 4 (1985): 814–820. 4029561 10.1016/0016-5085(85)90577-3

[11] J. Whitsett and R. C. Tsang , “In Vitro Effects of Fatty Acids on Serum‐Ionized Calcium,” Journal of Pediatrics 91, no. 2 (1977): 233–236. 874679 10.1016/s0022-3476(77)80818-4

[12] M. S. Cooper and N. J. L. Gittoes , “Diagnosis and Management of Hypocalcaemia,” BMJ 336, no. 7656 (2008): 1298–1302. 18535072 10.1136/bmj.39582.589433.BE PMC2413335

[13] G. Liamis , “Diabetes Mellitus and Electrolyte Disorders,” World Journal of Clinical Cases 2, no. 10 (2014): 488. 25325058 10.12998/wjcc.v2.i10.488 PMC4198400

